# Extraordinary therapeutic effect of PSMA radioligand therapy in treatment-refractory progressive metastatic prostate cancer with the transketolase inhibitor benfo-oxythiamine as a radiosensitizer—A case report

**DOI:** 10.3389/fmed.2024.1462234

**Published:** 2024-10-09

**Authors:** Carsten S. Kramer, Jingjing Zhang, Richard P. Baum

**Affiliations:** ^1^CURANOSTICUM Wiesbaden-Frankfurt, Center for Advanced Radiomolecular Precision Oncology, Wiesbaden, Germany; ^2^Department of Diagnostic Radiology, Clinical Imaging Research Centre, National University of Singapore, Singapore, Singapore

**Keywords:** radiosensitizer, transketolase, TKTL1, thiamine antagonist, benfo-oxythiamine, ^177^Lu-PSMA, metastatic castration-resistant prostate cancer, case report

## Abstract

Herein we report, for the first time, the therapeutic response of a prostate cancer patient with the thiamine antagonist benfo-oxythiamine (B-OT) added to prostate-specific membrane antigen (PSMA)-targeted radioligand therapy (PRLT). The patient was initially diagnosed as pT3b pN0 (0/7) M0 L0 V0 R0 G3, Gleason score 5 + 5 = 10, with an initial prostate-specific antigen (PSA) level of 4.05 ng/ml. Shortly after radical prostatectomy, ^68^Ga-PSMA positron emission tomography/computed tomography (PET/CT) revealed PSMA-positive lymph node metastases. Despite treatment with androgen deprivation therapy, external beam radiation therapy, palliative chemotherapy, and five cycles of PRLT (^177^Lu-PRLT or TANDEM-PRLT, respectively), the patient experienced progression in PSA levels as well as in PSMA PET/CT. Due to the intense PSMA expression, ^177^Lu-PRLT with ^177^Lu-PSMA-I&T was resumed for another 4 cycles (cycles 6th to 9th) and the patient was additionally treated with the thiamine antagonist benfo-oxythiamine. It was hypothesized that B-OT acts as a radiosensitizer by interfering with the repair of damaged DNA. B-OT-PRLT was well-tolerated and no substantial changes in laboratory results were observed. Additionally, the patient reported significant improvement in clinical symptoms. Post-treatment ^177^Lu-PSMA single-photon computed tomography (SPECT)/CT after the 7th cycle (and after 2 cycles of B-OT-PRLT) revealed regression of metastases compared to the post-treatment SPECT/CT after the 6th cycle. Before the 8th cycle, PSMA PET/CT showed a mixed response following prior uncontrollable cancer progression. Moreover, the PSA level showed a significant decline after one cycle of B-OT-PRLT. Although the patient had experienced massive progression before the first cycle of B-OT-PRLT, he survived for an additional 12 months. This case supports the hypothesis that B-OT-PRLT could overcome radiation resistance in prostate cancer patients who do not initially respond to ^177^Lu- or ^225^Ac-PRLT.

## 1 Introduction

The cell surface enzyme prostate-specific membrane antigen (PSMA) is highly expressed on prostate cancer cells, and that further correlates with the malignancy of the disease, whereas low or no PSMA expression is found on the surface of benign prostate cells (1, 2). These characteristics make PSMA a promising neoantigen for targeted radionuclide therapy for prostate cancer. PSMA-directed radioligand therapy (PRLT) with beta emitters (like lutetium-177, ^177^Lu) or alpha-emitters (like actinium-225, ^225^Ac) labeled PSMA ligands have demonstrated encouraging efficacy in metastatic castration-resistant prostate cancer (mCRPC) patients (3).

^177^Lu-PSMA radioligand therapy (^177^Lu-PRLT) is effective for the treatment of mCRPC, even in advanced cases, and lends a significant benefit to overall and progression-free survival (4). Significant PSA (prostate-specific antigen) decline by >50% was observed in 30–60% of the patients (4–9). According to early clinical experience, ^225^Ac-PSMA radioligand therapy has recently demonstrated remarkable therapeutic efficacy in heavily pre-treated mCRPC patients (10). Additionally, TANDEM-PRLT with ^225^Ac-PSMA/^177^Lu-PSMA has shown better treatment responses while minimizing severity of xerostomia in late-stage/end-stage mCRPC patients (11).

Despite the proven effectiveness of ^177^Lu-, ^225^Ac-PRLT, and TANDEM-PRLT, a substantial proportion of patients exhibit initial (or develop over the course) PRLT-refractory tumors despite high PSMA expression on PSMA PET/CT. As beta- or alpha-radiation induces cell death by the generation of single- and double-strand DNA breaks (12), progress under PRLT could be due to radiation resistance.

Transketolase enzymes play a crucial role in DNA repair and the formation of ribose-5-phosphate (R5P), an essential building block for the repair and formation of new deoxyribonucleic acid (DNA) strands. While the transketolase (TKT) is involved in DNA double-strand repair (13), the transketolase-like 1 transketolase (TKTL1) triggers ribose production by forming a TKT-TKTL1 heterodimer, ensuring sufficient building blocks are available for DNA synthesis and repair (14). Recent studies have shown that the product of TKTL1 fermentation metabolism (15), lactate, leads to a lactylation and activation of MRE11, an enzyme that facilitates the repair of the DNA strand breaks via homologous recombination (16). In summary, the importance of transketolases makes them a promising target for interfering with DNA repair in cancer cells, thereby increasing their sensitivity to radiotherapy. Recent research has provided initial insights into TKT-conferred radiation resistance in hepatocellular carcinoma (13).

The intrinsic dependence of transketolase on the tightly and quasi-irreversible bound cofactor thiamine(-pyrophosphate, vitamin B1) simplifies their inhibition as thiamine derivatives can act as suitable inhibitors (known as “thiamine antagonists”) (17, 18). The significant overexpression of transketolases in cancer cells not only serves as a marker for poor prognosis of cancer patients (19–21) but also provides a strategy for selective inhibition in cancerous rather than in healthy cells. Additionally, another factor contributing to the selectivity of thiamine antagonists against cancer cells arises from the quasi-irreversible binding mode of thiamine to transketolases: transketolases formed in the presence of thiamine bind it quasi-irreversibly, preventing the natural cofactor from being displaced by inhibitors. Therefore, only newly formed transketolases should be inhibited by thiamine antagonists. As cancer cells have a higher rate of transketolase expression, inhibitory thiamine derivatives should preferably affect transketolases in cancer cells and contribute to a broad therapeutic index. The exceptionally good safety profile of the inhibitory thiamine derivative benfo-oxythiamine (B-OT) was confirmed in a clinical phase 1 study with healthy volunteers (BV-01-101 / EudraCT number: 2021-005616-60). B-OT is a novel thiamine analog that acts as a prodrug and releases oxythiamine (OT), the actual inhibitor of thiamine pyrophosphate-dependent enzymes like transketolases.

Given the crucial role of transketolases in DNA production and repair, and their dependence on thiamine, it should be possible to influence DNA damage repair caused by radiotherapy with an inhibitory thiamine derivative such as B-OT. The survival of cancer cells depends on the proportion of radiation-induced DNA damage and the efficacy of DNA repair mechanisms. Therefore, a combination therapy of radioligand therapy and simultaneous DNA repair inhibition with B-OT would be advantageous. We hypothesized that combining B-OT with PRLT could reduce radiation resistance and achieve better therapeutic efficacy. A recent review summarizes current strategies to improve the efficacy of PRLT by combining it with other drug classes, such as immunotherapeutics or radiosensitizers like the isoflavone idronoxil or the PARP inhibitor olaparip (22). Phase I studies evaluating the combination of ^177^Lu-PSMA-617 with either idronoxil (23) or olaparib (24) have reported good safety and tolerability for both combinations. However, the data are currently insufficient to demonstrate a clear improvement in efficacy compared to single-agent radionuclide therapy.

Herein we report, for the first time, a case of exceptional response to ^177^Lu-PRLT combined with the radiosensitizer B-OT in a 79-year-old Caucasian man with mCRPC who progressed on all standard-of-care therapies as well as ^177^Lu-PRLT and TANDEM-PRLT.

## 2 Case presentation

### 2.1 Medical history and pretreatments

**Figure 1:**
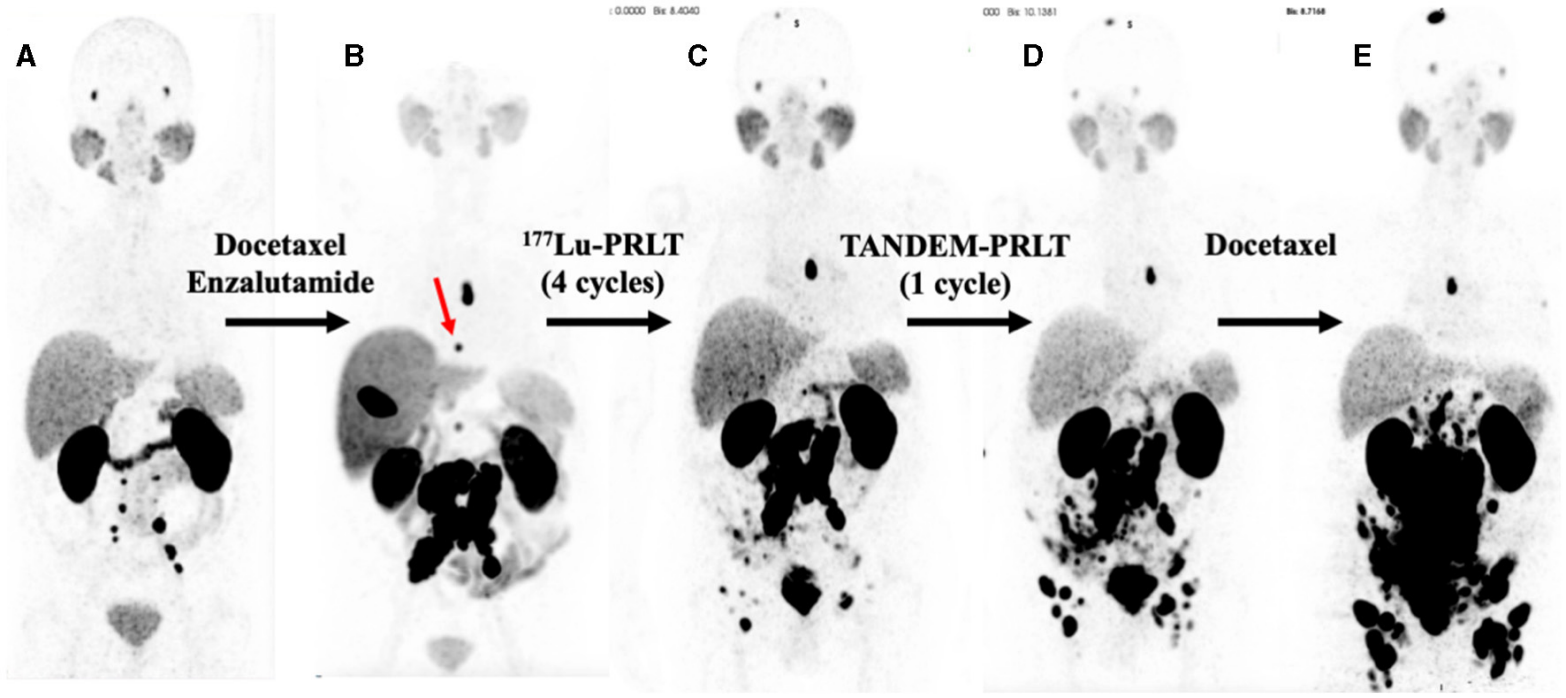
Serial PSMA PET maximum intensity projections (MIPs). **(A)** ^68^Ga-PSMA PET (2/2017), 2 years after diagnoses and prostatectomy, external beam radiation therapy, and treatment with leuprorelin. **(B)** ^18^F-PSMA PET (5/2019), after docetaxel chemotherapy and 1 year of enzalutamide exposure; red arrow: bone metastases in T8. **(C)** ^68^Ga-PSMA PET (11/2019) after four cycles of ^177^Lu-PRLT. **(D)** ^68^Ga-PSMA PET (1/2020) after one cycle of TANDEM-PRLT. **(E)** ^68^Ga-PSMA PET (5/2020) after six cycles of docetaxel chemotherapy.

**Figure 2:**
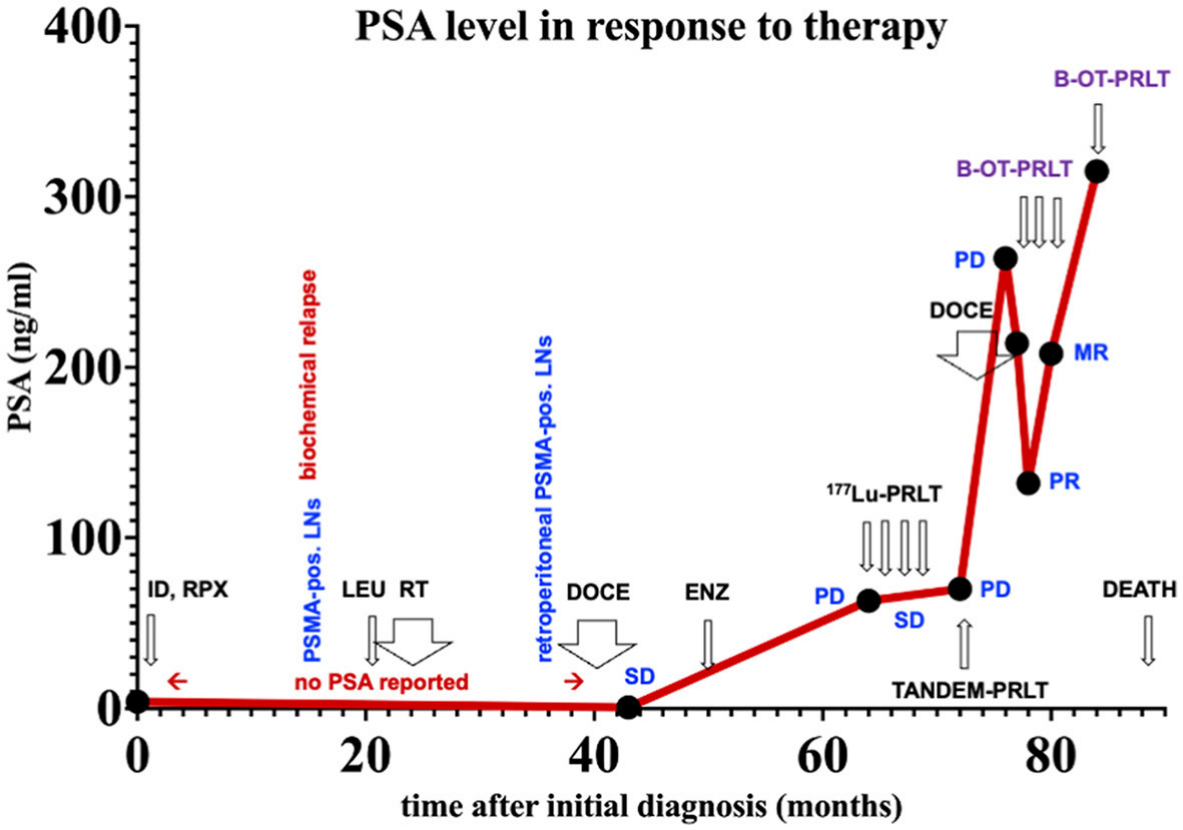
PSA values over time. ID, initial diagnoses; LNs, lymph nodes. Treatments: LEU, Leuprorelin; RT, external beam radiation therapy; DOCE, docetaxel; ENZ, enzalutamide; ^177^Lu-PRLT, ^177^Lu-PSMA RPLT; TANDEM-PRLT, ^177^Lu/^225^Ac-PRLT; B-OT-PRLT, ^177^Lu-PSMA PRLT combined with orally administered benfo-oxythiamine as radiosensitizer. Image findings: MR, mixed response; PD, progressive disease; PR, partial response; SD, stable disease.

A 79-year-old man (^*^1941) underwent a robot-assisted radical prostatectomy with lymphadenectomy for prostate cancer in Feb. 2015 after initial diagnosis in Dec. 2014. No relevant secondary diseases were reported. Initial tumor classification was pT3b pN0 (0/7) M0 L0 V0 R0 G3, Gleason score 5 + 5 = 10. The initial prostate-specific antigen (PSA) level in serum was 4.05 ng/ml. Three months after radical prostatectomy, the patient suffered from biochemical recurrence. ^68^Ga-PSMA positron emission tomography/computed tomography (PET/CT) revealed persistent primary prostate cancer with lymph node metastases. He received androgen deprivation therapy using the GnRH analog leuprorelin (Eligard^®^, start in Oct. 2015 and continued until death) and external beam radiation therapy (EBRT) of the prostate bed and the lymphatic drainage pathways (10–12/2015, total dose of 66.6 Gy). Two years later, ^68^Ga-PSMA PET/CT scan revealed multiple retroperitoneal lymph node metastases, but no organ involvement (Figure 1A). He underwent six cycles of palliative chemotherapy with docetaxel from (3–7/2017), achieving stable disease on ^68^Ga-PSMA PET/CT with a serum PSA nadir of 0.62 ng/ml (month 43 in Figure 2) followed by treatment with the androgen receptor antagonist enzalutamide (Xtandi^®^, start in May 2018). Despite 1 year of treatment with enzalutamide, he experienced disease progression (Figure 1B) with local recurrence and extensive lymph node metastases as well as appearance of a single bone lesion [red arrow in ^18^F-PSMA PET; since the lesion could be demarcated in subsequent ^68^Ga-PSMA scans and showed a correlate in CT, an unspecific bone uptake was unlikely (25)]; serum PSA increased to 63 ng/ml in May 2019.

Having progressed through all standard lines of therapy, the patient was treated with 2 cycles of ^177^Lu-PRLT (5–7/2019, cumulative activity 14.8 GBq of ^177^Lu), achieving stable disease on ^18^F-PSMA PET/CT in Aug. 2019. After an additional two cycles of ^177^Lu-PRLT (8–9/2019, cumulative activity of the 3rd and 4th cycle: 14.2 GBq of ^177^Lu), ^68^Ga-PSMA PET/CT (Figure 1C) in Nov. 2019 exhibited disease progression with extensive PSMA-avid lymph node and bone metastases; concurrent ^18^F-FDG PET/CT revealed increased glycolytic activity of the lymph node metastases. In Nov. 2019, the treatment was escalated to TANDEM-PRLT (6.2 GBq of ^177^Lu plus 2.1 MBq of ^225^Ac) but unfortunately, ^68^Ga-PSMA PET/CT performed in Jan. 2020 demonstrated further rapid lymphatic and osseous progression of the disease (Figure 1D) with a rising serum PSA level to 70 ng/ml.

The patient resumed docetaxel chemotherapy (six cycles from Feb. to May 2020, last cycle with reduced dose due to pancytopenia); however, ^68^Ga-PSMA PET/CT (Figure 1E) showed significant progression of the disease in May in 2020 with extensive, intense PSMA-positive inguinal, iliac, retroperitoneal, and mediastinal (infracarinal and retroesophageal) lymph node metastases, mesenteric and peritoneal involvement and infiltration of the pararectal and sigmoidal adipose tissue, intramural bladder wall infiltration as well as bone metastases. Treatment with enzalutamide was stopped due to PSA increase to 264 ng/ml.

## 3 Timeline of events

Figure 2 displays an overview of the timeline of events.

## 4 Treatment with B-OT-PRLT

Having progressed through all lines of standard therapy (Figure 2), as well as ^177^Lu-PRLT and TANDEM-PRLT, the patient was referred to our clinic. The patient reported fatigue, reduced performance, and diminished appetite (no xerostomia was present). Relevant secondary diagnoses included exertional dyspnea NYHA II, stress incontinence G3. The physical examination was unremarkable, except for alopecia and polyneuropathy after chemotherapy. The patient was treated with PRLT combined with B-OT as radiosensitizer (B-OT-PRLT) as follows: in 6/2020 7.4 GBq ^177^Lu-PSMA-I&T were administered (6th cycle) in combination with B-OT (3 mg/day for 5 days with oral dosing starting 2 days before PRLT). Before treatment, serum PSA level was 214 ng/ml; anemia G2 (Hb value 8.2 g/dl; normal > 13.5), and erythrocytopenia (2.76 million/μl; normal > 4.4), and leukocytopenia G2 (3.0 thousand/μl; normal > 3.8) were present, platelet count, creatinine and transaminases were within the normal range. Under leuprorelin, serum testosterone was completely suppressed (< 0.03 ng/ml, normal 1.93–7.40).

In July 2020, the patient received 8.6 GBq of ^177^Lu-PSMA-I&T (7th PRLT cycle) in combination with B-OT for 5 days (3 mg/day, starting 2 days before PRLT). Post-treatment SPECT/CT after the 7th cycle revealed excellent tumor regression (when compared to post-treatment SPECT/CT in 6/2022, Figure 3). Moreover, serum PSA declined by 50% from 264 to 132 ng/ml (5/2020 vs. 7/2020, Figure 2), or 38% within 8 weeks (early 6/2020 vs. late 7/2020) after one cycle of B-OT-PRLT. The patient reported a significant improvement of clinical symptoms and of his general condition and wellbeing. Laboratory analysis during the inpatient stay revealed anemia G1 (Hb 10.9 g/dl; normal > 13.5), erythrocytopenia (3.68 million/μl; normal > 4.4) with normal leukocyte and platelet counts. Serum creatinine, liver parameters, AP, LDH stayed within the normal range. Serum testosterone was at castration level.

**Figure 3:**
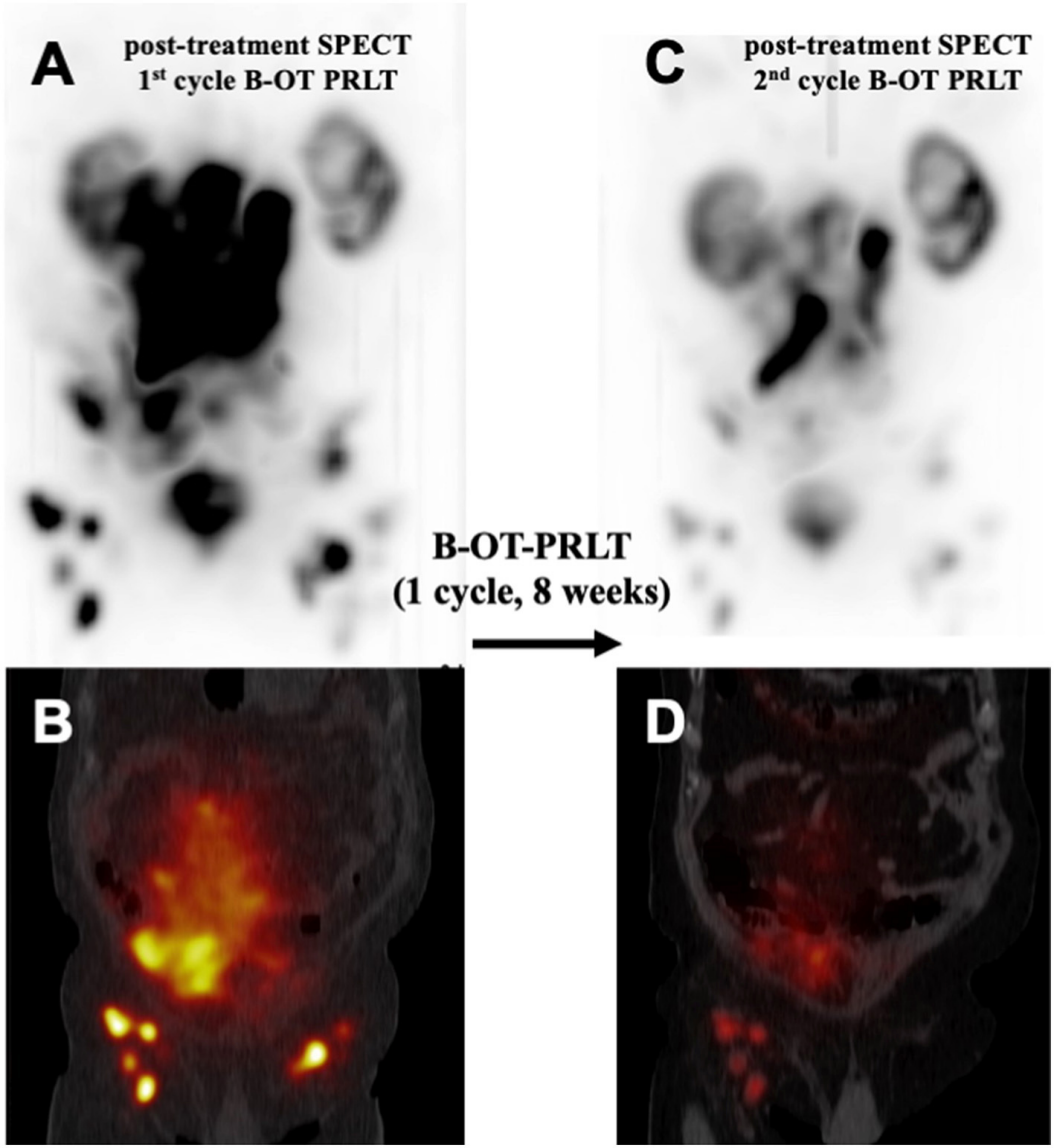
Comparison of post-treatment ^177^Lu-PSMA SPECT/CT examinations after one cycle of B-OT-PRLT: Post-treatment ^177^Lu-PSMA SPECT **(A)** and fused SPECT/CT image **(B)**, representing the 1st cycle of B-OT-PRLT in 6/2020. After 8 weeks, the post-treatment ^177^Lu-PSMA SPECT **(C)** and the fused SPECT/CT image **(D)** of the 2nd cycle of B-OT-PRLT in 7/2020 showed clearly partial remission of the disease.

**Figure 4:**
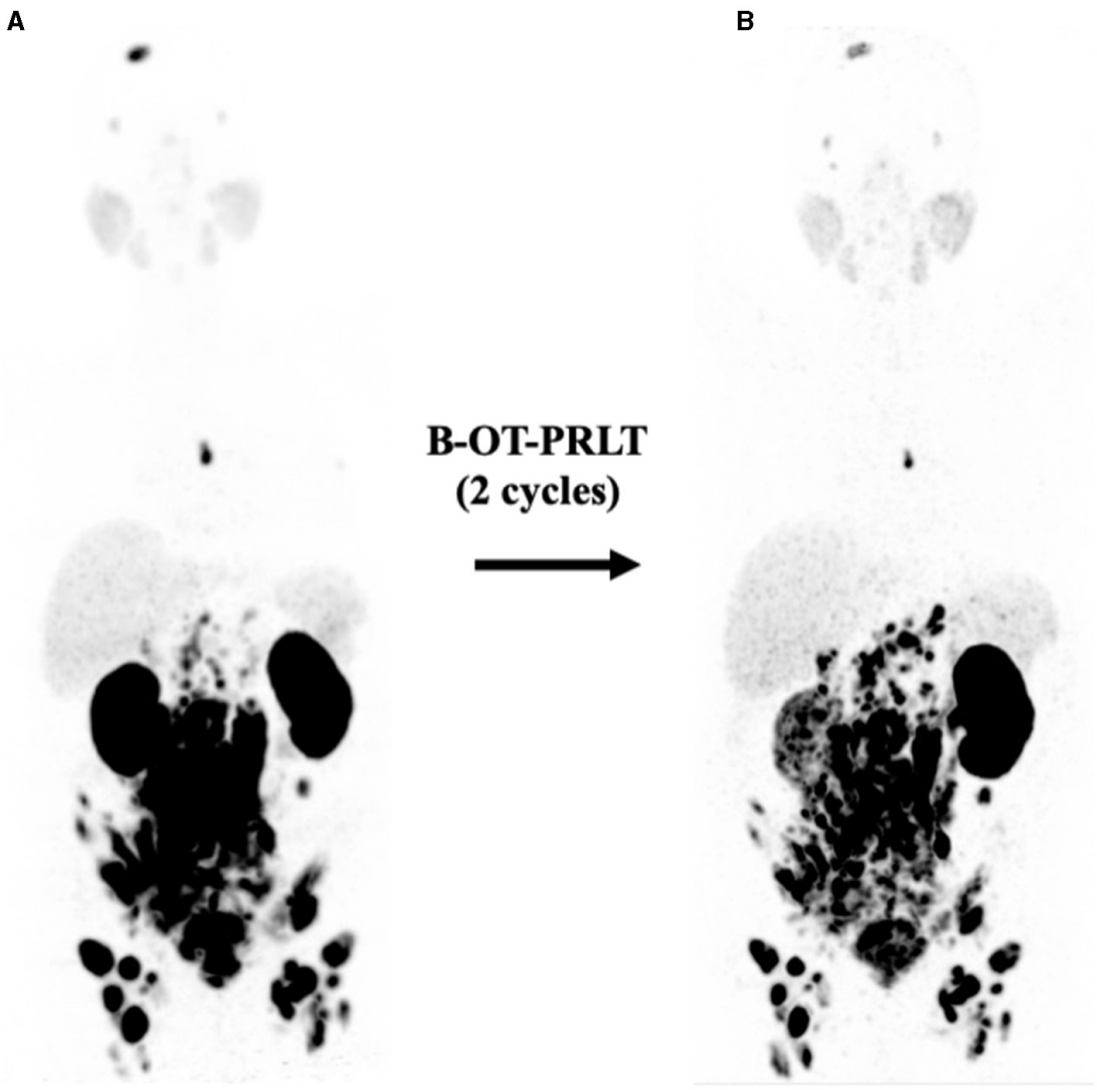
**(A)** Initial ^68^Ga-PSMA PET (MIP, 5/2020) after six cycles of docetaxel showing extensive disease (see text). **(B)** ^68^Ga-PSMA PET (MIP, 9/2020) revealed mixed response after two cycles of B-OT-PRLT.

In Sep. 2020, the patient received 8.3 GBq of ^177^Lu-PSMA-I&T (8th cycle) in combination with B-OT 3 mg/day for 2 days (starting 1 day before PRLT, reduced dose due to elevated creatinine). ^68^Ga-PSMA PET/CT performed shortly before the PRLT showed a mixed response after formally non-controllable disease progression (Figure 4). The serum PSA increased to 208 ng/ml.

Before PRLT, Hb dropped to 9.3 g/dl (normal > 13.5) and the patient received a transfusion of PRBC with improvement of Hb to 10.4 g/dl. Normal leukocyte and platelet count, albumin remained borderline decreased, transaminases and AP were normal, LDH was elevated (353 U/l, normal < 250). Due to tumor-related ureteral obstruction, creatinine was severely elevated (12.1 ng/ml) along with hyperkalemia (6.1 mmol/l), and hyperuricemia (10.8 mmol/dl). The patient underwent short-term dialysis and bilateral nephrostomy.

In Jan. 2021, before the 9th cycle of PRLT with 7.5 GBq of ^177^Lu-PSMA-I&T, ^68^Ga-PSMA PET/CT revealed disease progression of the bone and lymphatic metastases. The patient was (pre-)treated with 3 mg/d B-OT for 2 days (starting one day before RPLT) due to again elevated serum creatinine levels (resulting from urinary obstruction due to a dislocated nephrostomy). The PSA peaked to 315 ng/ml. Laboratory analyses revealed again anemia G2 (erythrocytes 2.5 million/μl; normal > 4.4; Hb 8 g/dl; normal > 13.7) after a total of eight ECs since 10/2020 and thrombocytopenia G1 (94 × 10^9^/l; normal > 163), leukocyte count was normal. Severely elevated creatinine (9.2 ng/ml), hyperkalemia (6.0 mmol/l), and hyperuricemia (10.2 mmol/dl) were present. Albumin was borderline decreased, transaminases, AP, and LDH were normal.

Although the patient had massive progression of the disease before the first cycle of B-OT-PRLT, the patient survived a further 12 months and deceased in May 2021. The patient experienced no xerostomia in context with the PRLT (nine cycles, total activity of 67 GBq ^177^Lu/2.1 MBq ^225^Ac), but reported weight loss of 10 kg in 4 months (76 kg, height 180 cm) due to reduced appetite after the 8th cycle of PRLT.

Other relevant diagnoses before death were chronic renal failure AKIN III with dialysis and macrohematuria, anemia G2 (treated with EC transfusions), thrombocytopenia G1, exertional dyspnea NYHA II-III, complete right bundle branch block, mild diastolic left ventricle dysfunction, arterial hypertension, and multiple renal cysts.

## 5 Discussion

The primary mode of action of external or internal radiotherapy is to cause DNA damage, thereby triggering the death of tumor cells. The success of this approach is determined by two key factors: the extent of DNA damage caused by the radiation and the degree to which this damage is repaired. Besides various genetically determined factors influencing repair ability, the availability of DNA building blocks also plays a crucial role. To enhance the vulnerability of cancer cells to DNA damage caused by radiation, we targeted the suppression of the transketolase enzyme. This approach reduces the availability of the DNA substrate R5P and inhibits DNA repair. By administering the novel thiamine antagonist and transketolase inhibitor B-OT, we aimed to enhance the effect of radioligand therapy in a radiation-resistant patient.

Unlike many other targets in oncology, the transketolase enzyme reaction is an essential metabolic pathway that cannot be replaced or bypassed by other metabolic pathways for the formation of ribose, which is crucial for the synthesis and repair of DNA strands. This makes the inhibition of the transketolase enzyme reaction an extremely effective strategy for disrupting DNA synthesis and repair. In addition to R5P synthesis and DNA repair, the TKTL1 transketolase also plays a central role in redox homeostasis and resistance against radiation (26, 32).

Consequently, in Werner's syndrome, a genetic mutation in the Werner protein causes the TKTL1 protein expression to be switched off (27), resulting in high levels of radicals and DNA damage, leading to a significantly shortened lifespan. For a long time, inhibiting transketolases in anticancer therapies was considered unfeasible due to the anticipated severe side effects, given that transketolases are ubiquitous and crucial enzymes of the pentose phosphate pathway, which ensures cell viability by supplying R5P. However, the tolerable and safe profile of B-OT, demonstrated in healthy volunteers in a recent phase I clinical study, suggests otherwise. In the cancer patient described here, no serious side effects attributable to B-OT were observed.

B-OT is a novel thiamine analog that acts as a prodrug, releasing OT, a compound metabolized into oxythiamine pyrophosphate. This metabolite inhibits thiamine pyrophosphate-dependent enzymes, such as transketolases (28). According to published nonclinical data, OT can significantly reduce R5P synthesis in cells and is considered a sensitizer for chemotherapies (21, 29, 30). Thiamine metabolism has been identified as a tumor-specific radiosensitizing pathway (31), and recent preclinical studies also indicate a role of TKTL1 in the acquisition of radioresistance (28, 32). In addition, tumor growth inhibition was observed in a prostate cancer LNCaP xenograft model after a combined therapy with B-OT and ^177^Lu-PSMA-617 supporting the hypothesis that B-OT is a radiosensitizer (personal information, unpublished data). Given these promising preclinical data, it was reasonable to offer the patient the option of co-treatment with B-OT, especially since TKTL1 expression is known to increase significantly during disease progression in prostate cancer patients (33). Without knowledge of the TKTL1 status of the subject, treatment with B-OT-PRLT showed exceptional effectiveness in the alpha and beta-radiation-refractory prostate cancer patient. Despite the patient showed progression even after TANDEM-PRLT, significant PSA decline and partial response/regression of metastases were observed with the co-administration of B-OT. Furthermore, the combination of ^177^Lu-PSMA and B-OT was very well-tolerated and the patient reported significant improvement in clinical symptoms, general condition, and wellbeing. Before the start of B-OT-PRLT, the patient was already anemic with reduced leucocyte levels due to previous treatment with docetaxel. However, over the course of B-OT-PRLT, no deterioration in hematological parameters was observed. While liver function remained unimpaired, later renal impairment was attributed to tumor-related urinary outflow obstruction.

Even though this case report documents only a single patient, the outcome is very encouraging. Our results provide initial evidence that combining ^177^Lu-PRLT with B-OT is a safe, feasible, and potentially effective therapeutic strategy for heavily pretreated patients with (acquired) radiation-refractory mCRPC. At the time the therapy was administered, data from the phase I clinical study with healthy volunteers, which showed that a daily dose of 5 mg over 7 days was well-tolerated, was not yet available. In this treatment trial, the daily administration of 3 mg over 5 days was significantly lower in both amount and duration than what was found to be tolerated in the phase I clinical study. Since our patient eventually progressed even under B-OT-PRLT, the daily dose of B-OT and the number of treatment days could potentially be increased, based on the safety data from the phase I study with healthy volunteers. To further evaluate the safety, tolerability, and efficacy of B-OT as a radiosensitizer, a phase 1b/2 open-label, single-center study will be conducted. The study will include both dose-escalation and dose-expansion phases. The results will help determine the optimal dosing regimen for B-OT when used in combination with various radioligand therapies.

## Data Availability

The raw data supporting the conclusions of this article will be made available by the authors, without undue reservation.
